# The impact of Medicaid expansion on coverage among those lacking housing basics, 2010-2019

**DOI:** 10.1093/haschl/qxag115

**Published:** 2026-06-04

**Authors:** Chengcheng Zhang, Deborah Freund, Gerald F Kominski, Petra W Rasmussen

**Affiliations:** Department of Health Sciences, Towson University, Towson, MD 21252, United States; Fielding School of Public Health, University of California, Los Angeles, CA 90095, United States; Fielding School of Public Health, University of California, Los Angeles, CA 90095, United States; Rand Corporation, Santa Monica, CA 90401, United States

**Keywords:** ACA, Medicaid expansion, housing conditions, health insurance disparities, social determinants of health, I13, I12

## Abstract

**Introduction:**

Housing is a critical social determinant of health, and individuals living in substandard housing often face greater healthcare needs and financial burdens. Expanding health insurance is therefore especially important for this population. However, little is known about whether the Affordable Care Act's Medicaid expansion affected disparities in coverage by housing quality. We examined whether Medicaid expansion improved insurance coverage equitably among low-income individuals living in homes with and without basic housing necessities.

**Methods:**

Using nationally representative survey data from 2010-2019, we compared changes in insurance coverage before and after expansion in states that expanded Medicaid eligibility vs those that did not.

**Results:**

Medicaid expansion increased Medicaid coverage by more than 12% points and reduced uninsurance by 6%-7% points relative to non-expansion states among individuals lacking at least one basic housing necessity. However, compared with individuals with complete housing necessities, those lacking at least one basic housing necessity experienced slightly smaller increases in Medicaid coverage and smaller reductions in uninsurance.

**Conclusion:**

Medicaid expansion improved coverage among individuals facing housing-related disadvantage but did not eliminate disparities. Eligibility-based expansions can extend coverage to structurally disadvantaged populations, but additional efforts to address non-financial barriers may be needed.

## Introduction

As a central component of the Affordable Care Act (ACA), Medicaid expansion was designed to increase health insurance coverage among low-income adults by extending eligibility to individuals with incomes up to 138% of the federal poverty level (FPL).^1-6^ Following the 2012 Supreme Court decision in *National Federation of Independent Business* vs *Sebelius* on the constitutionality of the ACA, states were allowed to decide whether to adopt Medicaid expansion. As of 2026, 41 states and the District of Columbia have adopted it, while 9 have not. Medicaid expansion has significantly increased coverage for low-income populations who previously lacked access to employer-sponsored or public coverage.^7,8^ This is reflected in the uninsured rate in the country—as of 2024, 8.2% of the US population were uninsured, down from 14.6% prior to the implementation of the ACA's coverage expansions in 2013.^9^

Substandard housing is common among low-income households and is associated with greater health needs and higher reliance on affordable insurance.^10-17^ Individuals lacking basic housing necessities may face competing demands and institutional barriers that complicate interaction with insurance systems, even when coverage is available at low or no cost. These considerations underscore the importance of examining whether eligibility-based coverage expansions are effective in reaching populations facing non-financial structural disadvantage.

A growing body of literature has documented the effects of Medicaid expansion on insurance coverage across a range of vulnerable populations using quasi-experimental designs.^2-7,18-28^ However, housing quality, a fundamental social determinant of health, has been largely overlooked in causal coverage studies^29,30^ and few studies have examined whether housing quality limited access to coverage gains under Medicaid expansion. Prior descriptive work shows that individuals lacking basic housing necessities have lower insurance coverage rates,^16^ but does not isolate the effect of Medicaid expansion or test whether housing-related constraints limited access to coverage gains. Although Medicaid expansion directly reduces financial barriers to coverage, lack of basic housing necessities may capture additional non-financial structural constraints, such as administrative burden and limited access to communication infrastructure, that could affect enrollment and retention among income-eligible individuals. This study uses quasi-experimental methods to assess whether Medicaid expansion impacts individuals who lack basic housing necessities.

This study makes several important contributions to the literature on Medicaid expansion and health insurance coverage. First, it addresses a critical gap by assessing whether Medicaid expansion effectively reached individuals lack of basic housing necessities, a population facing substantial non-financial structural disadvantage. Second, we leverage a decade of nationally representative data and apply rigorous quasi-experimental methods, and conduct extensive sensitivity analyses to estimate credible causal effects of Medicaid expansion on insurance coverage. Third, our findings provide evidence that Medicaid expansion increased coverage among individuals living in homes with and without basic housing necessities. There is modest evidence that individuals lacking housing necessities experienced slightly smaller increases in Medicaid coverage. These findings highlight the capacity of eligibility-based expansions to promote equitable access to insurance despite persistent structural vulnerability. Overall, these results inform policy discussions about the effectiveness of eligibility-based coverage expansions in reaching populations often considered hard to reach.

## Data

We use data from the U.S. Census Bureau's American Community Survey (ACS) from 2010-2019, a nationally representative annual survey that includes detailed information on health insurance coverage, housing characteristics, and sociodemographic factors. We restrict the analytic sample to adults aged 18-64 with household income at or below 138% of the FPL, consistent with Medicaid expansion eligibility, and residing in non-homeless households with varying levels of access to basic housing necessities.

### Measures

Our primary outcomes are binary indicators for four types of health insurance coverage:

(i) being uninsured, (ii) having Medicaid coverage, (iii) having employer-sponsored insurance, and (iv) having individual purchased private insurance coverage. These are derived from ACS self-reports of insurance status at the time of the survey.

We construct a measure of housing quality using basic housing necessities derived from ACS housing characteristics and use this measure to classify individuals based on whether they lack at least one basic housing necessity. Basic necessities include access to a bathtub or shower, heating, a sink with running water, a stove or range, telephone service, and a refrigerator.

Models adjust for age measured in years and sex (male or female). Race and ethnicity are categorized into six groups based on ACS classifications and are included to account for structural and socioeconomic differences associated with both housing conditions and health insurance coverage, which may confound the estimated effects of Medicaid expansion. Educational attainment is grouped into four categories: less than high school, high school graduate, some college, and college or above. Marital status is defined as married vs not married. Employment status is categorized into four groups: employed, unemployed, not in the labor force, and other. Citizenship status is defined as U.S. citizen vs non-citizen. Household income is included as a log-transformed continuous variable, and family size is measured as the number of individuals in the household. All models additionally include state and year fixed effects.

To analyze the impact of Medicaid expansion, states are classified based on expansion status as of 2015. An expansion indicator equals 1 if the state enacted ACA Medicaid expansion in 2015 or earlier, and 0 otherwise. To avoid heterogeneity in expansion timing, we exclude six states that expanded Medicaid eligibility between 2015 and 2020 (Alaska, Indiana, Louisiana, Maine, Montana, and Virginia). Including these late expansion states would complicate the interpretation of pre- and post-expansion comparisons in a difference-in-differences (DID) framework. The final analytic sample includes 27 expansion states and the District of Columbia, and 17 non-expansion states (Appendix Table 1).

## Study design

### Main analysis

We estimate the effects of Medicaid expansion on health insurance coverage using a DID approach that compares changes in coverage before (2010-2013) and after (2015-2019) Medicaid expansion between expansion and non-expansion states, treating 2014 as a transition year. This approach isolates changes in insurance coverage associated with Medicaid expansion by contrasting trends in states that expanded Medicaid with those that did not.

To assess whether coverage gains differed by housing-related disadvantage, we extend this framework using a triple-difference (DDD) approach. This model compares changes over time between individuals living in homes with and without access to basic housing necessities in expansion vs non-expansion states. This DDD estimates capture whether Medicaid expansion had differential effects for individuals facing greater non-financial structural disadvantage related to lack at basic necessities. Formal model specifications are provided in Appendix A.

### Robustness checks

We conduct a series of sensitivity analyses to assess the robustness of our findings. First, to account for potential heterogeneity in treatment timing, we restrict the sample to states that expanded Medicaid in 2014 and compare them with states that did not expand during the study period. We exclude early expansion states (California, Massachusetts, Minnesota, Washington, and the District of Columbia) as well as states that expanded after 2014 (Pennsylvania, Alaska, Indiana, Louisiana, Maine, Montana, and Virginia). This specification ensures that all treated states adopt Medicaid expansion at the same time (2014), improving the validity of the DID comparison.

Second, given the relatively small number of state clusters, we re-estimate all models using wild cluster bootstrap methods at the state level.

Third, we test whether our findings are sensitive to alternative housing measures using DDD specifications. We create several alternative measures of housing disadvantage, including grouped infrastructure deficits (ie, missing bathtub, sink, heating, or stove), communication barriers (ie, lack of telephone service), storage deficit (ie, refrigerator) and measures capturing the number of missing necessities. Additionally, we conduct a DDD analysis based on housing tenure (renting vs homeownership) to examine whether the effects of Medicaid expansion differ across broader housing circumstances.

Fourth, we restrict the analytic sample to adults aged 26-64 to account for other ACA provisions, such as dependent coverage for young adults, that may affect insurance coverage independently. Finally, we assess the parallel trends assumption using event study models and test whether pre-expansion trends differ between expansion and non-expansion states.

## Results

### Demographic characteristics

Table 1 presents descriptive statistics for the pre-expansion period (2010-2013), allowing us to compare baseline characteristics across groups defined by access to basic housing necessities prior to Medicaid expansion. For many socioeconomic characteristics, individuals whose homes lack one or more basic necessities are at a noticeable disadvantage compared to those homes with full necessities. For example, annual household incomes for those whose homes lack at least one basic necessity are approximately 2500 dollars lower than those with complete necessities. Some other observations include differences in marital status and racial composition. Among individuals who live at home lacking at least one basic necessity, 25.3% are married vs 29.2% of those with complete necessities. Racial composition also differs across groups defined by housing necessity status. White individuals make up a larger share of those living in homes with complete necessities (64.4%) than those lacking housing basics (61.5%). In contrast, several minority groups are relatively more prevalent among individuals living in substandard housing, indicating disproportionate representation in more disadvantaged housing conditions.

**Table 1.** Descriptive statistics.

Variable	Full sample (sample size: 9, 529, 361)	Housing quality	
		Lacking at least one basic necessity (sample size: 344, 007)	With complete basic necessities (sample size: 9, 185, 354)
Age	36.4	35.7	36.4
	(12.6)	(12.4)	(12.6)
Household income	24, 305.8	21, 991.7	24, 449.7
	(26, 727.0)	(22 000.6)	(26 987.1)
Family size	3.8	3.9	3.8
	(1.8)	(2.0)	(1.8)
Unemployed (%)	14.8	15.3	14.8
Married (%)	28.5	25.3	29.2
Sex (%)			
Female	56.2	52.2	56.9
Male	43.8	47.8	43.1
Race (%)			
American India/Alaska Native/Asian/Native Hawaiian/	0.8	1.2	0.7
Pacific Island	4.8	6.1	4.8
Black	19.5	18.2	19.4
White	64.1	61.5	64.4
Multiracial	2.6	3.3	2.5
Other	8.2	9.8	8.2
Any health insurance (%)			
Uninsured	42.2	46.8	42.1
Medicaid	31.9	32.8	31.3
Employer-sponsored	19.6	15.1	20.1
Directly-purchased	6.3	5.3	6.5

Estimates are based on the ACS 2010-2013. The sample is restricted to adults aged 18-64 with family income at or below 138% of the federal poverty level and excludes individuals covered by Veterans Affairs health care or Indian Health Service. All estimates are weighted using ACS person-level survey weights. Percentages are reported for categorical variables, and means are reported for continuous variables (age, household income, and family size), with standard deviations in parentheses.

Before the ACA went into effect, individuals who lived in homes lacking at least one basic necessity were more likely to be uninsured (46.8% vs 42.1%). In addition, they are more likely to be covered by Medicaid (32.8% vs 31.3%), and less likely to be covered by employer-sponsored (15.1% vs 20.1%) or directly purchased private insurance (5.3% vs 6.5%) than those with complete housing necessities. These differences underscore the structural vulnerability of individuals lacking basic housing necessities prior to Medicaid expansion.

### Coverage changes by expansion status

Table 2 presents unadjusted changes in insurance coverage by expansion status and access to basic housing necessities, along with adjusted DID and DDD estimates for individuals with incomes up to 138% FPL. In both expansion and non expansion states, the percentage of uninsured individuals living in homes lacking at least one basic necessity decreased over time. During the same period, Medicaid coverage increased.

**Table 2.** Difference-in-differences and triple-difference estimates of insurance coverage changes.

		Expansion (%)		Non-expansion (%)				Triple difference (95% CI)	
Outcome	Housing quality group	Pre-expansion 2010-2013	Post-expansion 2015-2019	Pre-expansion 2010-2013	Post-expansion 2015-2019	Adjusted DID (95% CI)	*P*-value		*P*-value
Uninsured	With complete basics	36.3	17.3	46.7	33.7	−6.6 (−9.7, −3.6)	0.000	2.9 (0.1, 5.7)	0.040
	Lacking at least one	41.5	23.3	51.3	39.0	−6.0 (−10.2, −1.8)	0.006		
Medicaid	With complete basics	34.4	51.1	23.7	27.7	13.1 (10.2, 16.1)	0.000	−3.0 (−5.0, −1.0)	0.005
	Lacking at least one	34.6	51.0	26.6	30.8	13.3 (9.3, 17.2)	0.000		
Employer-insured	With complete basics	20.8	21.9	20.6	24.1	−2.7 (−3.5, −1.9)	0.000	1.4 (0, 2.8)	0.055
	Lacking at least one	16.5	16.9	14.2	17.8	−3.6 (−5.3, −2.0)	0.000		
Directly purchased	With complete basics	8.5	10.3	7.4	12.3	−4.8 (−6.3, −3.2)	0.000	0.7 (−0.3, 1.8)	0.181
	Lacking at least one	7.1	9.4	6.5	11.3	−5.0 (−8.2, −1.7)	0.003		

Estimates are from survey-weighted linear probability models. Coefficients represent percentage point changes. Models include demographic controls, state fixed effects, and year fixed effects. All estimates are weighted using ACS person-level survey weights. The sample is restricted to adults aged 18-64 with income ≤138% FPL. 2014 is excluded as a transition year.

Adjusted DID results show that Medicaid expansion significantly increased coverage and reduced uninsurance across groups defined by access to basic housing necessities. Among lacking at least one basic necessity, Medicaid coverage increased by 13.3% points more in expansion states than in non-expansion states (95% CI, 9.3 to 17.2), while the uninsurance rate declined by 6% points more (95% CI, −10.2 to −1.8). Meanwhile, changes in private coverage were larger in non-expansion states, with employer-sponsored insurance increasing by 3.6% points more (95% CI, −5.3 to −2.0) and directly purchased insurance increasing by 5 (95% CI, −8.2 to −1.7) percentage points more relative to expansion states.

DDD estimates show differential effects by access to basic housing necessities. The increase in Medicaid coverage is 3% points smaller among individuals lacking at least one basic housing necessity than among those with complete necessities (95% CI, −5.0 to −1.0). Similarly, the decline in uninsurance is 2.9% points smaller among individuals lacking at least one basic housing necessity than among those with complete necessities (95% CI, 0.1 to 5.7). For employer-sponsored and directly-purchased insurance, the results are not statistically significant, indicating that coverage gains were broadly similar between individuals with and without basic housing necessities.

**Figure 1.**
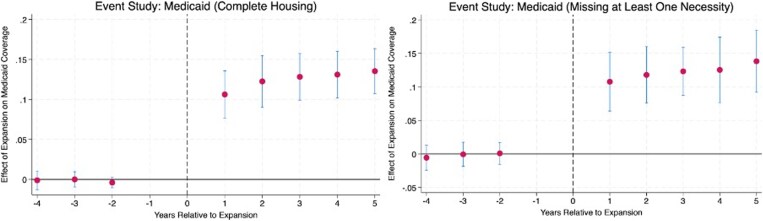
Event study estimates of the effect of Medicaid expansion on Medicaid coverage by housing quality. Estimates are obtained from survey-weighted linear probability models that include individual-level covariates, state fixed effects, and year fixed effects. Coefficients are normalized to the year prior to expansion. Error bars represent 95% confidence intervals. The sample is restricted to adults ages 18-64 with family income at or below 138% of the federal poverty level. States that expanded Medicaid before or after 2014 are excluded.

We find no evidence of differential pre-trends for uninsured, Medicaid, or employer-sponsored coverage, supporting the parallel trends assumption for the main outcomes (Figure 1). Following Medicaid expansion, we observe immediate and sustained increases in Medicaid coverage and declines in uninsurance, along with reductions in employer-sponsored and directly purchased coverage. Although estimates for directly purchased coverage show some evidence of differential pre-trends in linear trend tests (Figure S1).

### Robustness checks

Table 3 presents results from a sensitivity analysis restricting the sample to states that expanded Medicaid in 2014 and states that did not expand during the study period. Our results are highly consistent with our main findings. Among individuals with complete housing necessities, Medicaid expansion increased Medicaid coverage by 12.6% points (95% CI, 9.3 to 15.8) and reduced uninsurance by 6.3% points (95% CI, −9.5 to −3.1). Similar effects are observed among individuals lacking housing necessities, with Medicaid coverage increasing by 11.8% points (95% CI, 8.3 to 15.3) and uninsurance declining by 5.2% points (95% CI, −9.0 to −1.4). Patterns for employer-sponsored and directly purchased coverage are also consistent with the main analysis. These results confirm that our findings are not driven by variation in the timing of Medicaid expansion and are robust to restricting the sample to a single adoption cohort.

**Table 3.** Robustness check: 2014 expansion states only difference-in-differences estimates.

Outcome	With complete basics adjusted DID (95% CI)	Lacking at least one adjusted DID (95% CI)
Uninsured	−6.3*** (−9.5, −3.1)	−5.2*** (−9.0, −1.4)
Medicaid	12.6*** (9.3, 15.8)	11.8*** (8.2, 15.3)
Employer sponsored	−2.8*** (−3.7, −1.8)	−2.9*** (−4.6, −1.2)
Direct purchase	−3.3*** (−4.8, −1.8)	−3.4*** (−5.6, −1.2)

Estimates are from survey-weighted linear probability models. Coefficients represent percentage point changes. Models include demographic controls, state fixed effects, and year fixed effects. All estimates are weighted using ACS person-level survey weights. The sample is restricted to adults aged 18-64 with income ≤138% FPL. 2014 is excluded as a transition year. *** *P* < 0.01.

To address potential bias in inference arising from a limited number of state clusters, we re-estimate the models using wild cluster bootstrap methods at the state level. The results are robust to this alternative inference approach: DID estimates remained statistically significant across outcomes and housing groups, whereas DDD estimates remained small and are not statistically significant (Appendix Table S2).

We further assess the robustness of our findings using alternative measures of housing disadvantage (Appendix Table S3). Overall, the DDD estimates are small in magnitude and not statistically significant across most outcomes, indicating that the main findings are not sensitive to how housing disadvantage is measured. Lack of telephone access is associated with a smaller increase in Medicaid coverage and a larger increase in directly purchased insurance, suggesting that communication-related constraints may shape insurance enrollment. Some specific housing problems, such as lack of a stove or sink, are statistically related to changes in insurance outcomes, although these patterns were not consistent across outcomes.

In Appendix Table S3, Panel D, renters experience a 2.1-percentage-point smaller increase in Medicaid coverage than homeowners (95% CI, −3.5 to −0.8).

Appendix Table S4 shows that the results are also robust to restricting the sample to adults aged 26-64. Medicaid expansion continues to significantly reduce uninsurance and increase Medicaid coverage across both housing groups, with effect sizes similar to the main analysis. For example, Medicaid coverage increases by approximately 13.7% points and uninsurance declines by 6%-7% points in both groups. Patterns for employer-sponsored and directly purchased insurance are also consistent with the main results. These findings indicate that the results are not driven by ACA-dependent coverage provisions affecting younger adults.

## Discussion

This study examines whether Medicaid expansion under the ACA increased insurance coverage among low-income individuals living in homes with and without basic housing necessities. Using nationally representative data and quasi-experimental methods, we find that Medicaid expansion substantially increased Medicaid coverage and reduced uninsurance among individuals with incomes up to 138% of FPL. Importantly, we find modest evidence that individuals lacking basic housing necessities experienced smaller increases in Medicaid coverage and smaller reductions in uninsurance.

These findings are notable because individuals lacking basic housing necessities typically have higher uninsurance rates before the expansion,^16,29^ reflecting broader structural disadvantage. They may also face additional barriers to enrollment such as limited access to communication (eg, no telephone service) or difficulty navigating administrative processes, consistent with prior literature on behavioral and administrative barriers to program participation.^31,32^ Despite these challenges, Medicaid expansion appears to have reached a population that is often considered difficult to engage through insurance expansions, suggesting that eligibility-based expansions can extend coverage to structurally disadvantaged groups.

At the same time, disparities in coverage persist after the expansion. Individuals lacking basic housing necessities remain more likely to be uninsured and experience smaller increase in Medicaid. These findings indicate that eligibility expansions alone may be insufficient to eliminate structural gaps in coverage and that non-financial barriers associated with housing conditions may continue to limit the extent of coverage gains.

Despite these barriers, several features of ACA implementation may have helped expand coverage among individuals facing housing-related disadvantage. Simplified enrollment and renewal processes, outreach through navigators and community-based organizations, and interactions with safety-net providers may have reduced administrative and informational barriers to enrollment. These mechanisms are not directly tested in our study but are consistent with prior evidence on program participation among disadvantaged populations.

Our findings have important implications for ongoing policy debates. While Medicaid expansion appears effective in increasing coverage among structurally disadvantaged populations, persistent gaps suggest that eligibility expansions alone are insufficient to eliminate disparities. Policies that reduce administrative burden and improve outreach to individuals facing housing-related disadvantage may be necessary to achieve more equitable coverage outcomes.

This study has several limitations. First, our analysis focuses on insurance coverage and does not examine downstream health outcomes. Second, the ACS does not capture broader housing or neighborhood conditions. Future research could link ACS data with neighborhood-level indicators such as crime rates, school quality, transportation access, or housing prices. These characteristics may help explain geographic variation in insurance access and Medicaid participation. In addition, further work is needed to identify the mechanisms underlying the observed reductions in uninsurance among individuals lacking basic housing necessities.

## Conclusion

Medicaid expansion under the ACA substantially increased insurance coverage among low-income individuals, including those living in homes lacking basic housing necessities. However, individuals lacking basic housing necessities experienced slightly smaller increases in Medicaid coverage and smaller reductions in uninsurance than those with complete housing necessities, indicating persistent disparities in coverage. These findings suggest that Medicaid expansion can meaningfully reduce coverage gaps among structurally disadvantaged populations, but does not fully eliminate differences associated with housing-related disadvantage. As such, eligibility-based expansions improve access to coverage but may need to be complemented by policies that address non-financial barriers to achieve more equitable outcomes.

## Supplementary Material

qxag115_Supplementary_Data
