# Supplementary material for: The impact of Medicaid expansion on coverage among those lacking housing basics, 2010-2019
Source: Health Aff Sch. 2026 Jun 4;4(6):qxag115. doi: 10.1093/haschl/qxag115 (PMC13234947; doi:10.1093/haschl/qxag115)
Supplement: qxag115_Supplementary_Data [file qxag115_supplementary_data.zip › 0.Appendix_resubmit2.docx]

**Appendix**

1. **Model Specification**

**Regression Equation – Adjusted model**

*Y_i_* = *β*_0_ + *β*_1_*X_i_* + *β*_2_*Y ear*_*after*2014*_t_ × Expansion*_*in*_*Effect_st_*

(1)

+ *β*_3_*Y ear*2014*_t_ × Expansion*_*in*_*Effect_st_* + Ω *Year_i_* + *µState_s_* + *ϵ_i,s,t_*

where *Y_i,s,t_* refers to *Uninsured_i,s,t_*, *Medicaid_i,s,t_* and *Private_i,s,t_* which stand for the per-centage of coverage for individual i in s state in year t. *X_i_* is a vector of covariates (household income, age, sex, education, race, employment status, citizenship and marital status). Ω is a vector of year fixed effects, and *µ* is a vector of state fixed effects. *β*_2_ represents the difference-in-differences estimate for comparing health insurance coverage rates pre (2010- 13) versus post (2015-19) implementation of the ACA between Medicaid expansion and non-expansion states among individuals occupy various quality housing. *ϵ_i,s,t_* is the error term. To assess whether Medicaid expansion effects differed by housing quality, we estimate the following triple-differences specification:

**Difference-in-differences-in-differences equation:**

*Y_i_* = *β*_0_ + *β*_1_*X_i_* + *β*_2_*Year*_*after*2014*_t_ × Expansion*_*in*_*Effect_st_*

(2)

+ *β*_3_*Y ear*2014*_t_ × Expansion*_*in*_*Effect_st_*

+ *β*_4_*Y ear*2014*_t_ × Expansion*_*in*_*Effect_st_ × Lacking_i_* + Ω*Y ear_i_* + *µState_s_* + *ϵ_i_*

where *Y_i,g,s,t_* now represents a binary insurance coverage outcome for individuals living in states in lacking necessity category g in year t. *Lacking_i_* represents the subgroup of interest which contains two levels. It equals to 1 if the individuals live in houses missing at least one basic necessity and 0 otherwise. The coefficient of our DDD indicator *β*_4_ estimates the additional impact of Medicaid expansion for those living in houses lacking at least one basic necessity. The magnitude of *β*_4_ indicates how much bigger the pre-post change in insurance coverage is for those lacking at least one vs. with complete necessities living in expansion states compared to the pre-post change for those lacking at least one vs. with those with complete necessities living in non-expansion states.

1. **State Expansion Classification**

Table A1: Status of State Action on the Medicaid Expansion Decision

| **Expansion Status** | **States** |
| --- | --- |
| Expanded before 2014 | California, District of Columbia, Massachusetts, Minnesota, Washington |
| Expanded in 2014 | Arizona, Arkansas, Colorado, Connecticut, Delaware, Hawaii, Illinois, Iowa, Kentucky, Maryland, Michigan, Nevada, New Hampshire, New Jersey, New Mexico, New York, North Dakota, Ohio, Oregon,  Rhode Island, Vermont, West Virginia |
| Expanded after 2014 | Alaska (2015), Indiana (2015), Pennsylvania (2015), Louisiana (2016), Montana (2016), Maine (2019), Virginia (2019) |
| Did not expand during study period (2015-2019) | Alabama, Florida, Georgia, Idaho, Kansas, Mississippi, Missouri, Nebraska, North Carolina, Oklahoma,  South Carolina, South Dakota, Tennessee, Texas, Utah, Wisconsin, Wyoming |

Sources: Kaiser Family Foundation

Alaska implemented expansion on 9/1/2015. Indiana implemented on 2/1/2015. Louisiana implemented on 7/1/2016. Maine implemented on 1/10/2019. Montana expansion on 1/1/2016. Virginia implemented 1/1/2019.

1. **Sensitivity Analyses**

Table A2. Wild Cluster Bootstrap Inference for Medicaid Expansion Effects

| **Outcome** | **Housing group** | **DID Coefficient**  **(95% CI)** | **Bootstrap**  **p-value** | **DDD Coefficient**  **(95% CI)** | **Bootstrap**  **p-value** |
| --- | --- | --- | --- | --- | --- |
| **Uninsured** | With complete basics | -0.067  (-0.098, -0.036)) | <0.001 | 0.001  (-0.020, 0.021) | 0.957 |
|  | Lacking ≥1 necessity | -0.066  (-0.112, -0.021) | 0.020 |  |  |
| **Medicaid** | With complete basics | 0.125  (0.095, 0.156) | <0.001 | -0.006  (-0.022, 0.011) | 0.518 |
|  | Lacking ≥1 necessity | 0.122  (0.086, 0.158) | <0.001 |  |  |
| **Employer-sponsored** | With complete basics | -0.024  (-0.033, -0.016) | <0.001 | 0.002  (-0.010, 0.014) | 0.772 |
|  | Lacking ≥1 necessity | -0.024  (-0.039, -0.008) | 0.012 |  |  |
| **Direct purchase** | With complete basics | -0.031  (-0.046, -0.017) | <0.001 | 0.003  (-0.007, 0.013) | 0.556 |
|  | Lacking ≥1 necessity | -0.027  (-0.049, -0.006) | 0.003 |  |  |

Notes: Each cell reports coefficients from linear probability models. Wild cluster bootstrap p-values are computed at the state level with 9,999 replications using Rademacher weights. Models include the same controls, state fixed effects, and year fixed effects as the main specification. 2014 is excluded as a transition year.

**Table A3. Sensitivity analyses using alternative measures of housing disadvantage** (triple-difference estimates from survey-weighted linear probability models, reported as coefficient (95% CI).

| **Housing dimension** | **Uninsured** | **Medicaid** | **Employer** | **Direct purchase** |
| --- | --- | --- | --- | --- |
| **Panel A. Grouped domains** |  |  |  |  |
| Infrastructure deficit | -0.005  (-0.032, 0.022) | 0.003  (-0.023, 0.028) | 0.003  (-0.013, 0.020) | -0.007  (-0.022, 0.008) |
| Telephone access  deficit | 0.014  (-0.005, 0.032) | -0.022**  (-0.040, -0.005) | 0.001  (-0.013, 0.015) | 0.009**  (0.000, 0.018) |
| Refrigerator deficit | -0.042  (-0.087, 0.003) | -0.024  (-0.071, 0.022) | 0.034  (-0.008, 0.075) | 0.022  (-0.008, 0.053) |
| **Panel B. Intensity measures** |  |  |  |  |
| Number of missing  necessities | -0.003  (-0.014, 0.009) | -0.007  (-0.017, 0.002) | 0.004  (-0.006, 0.014) | 0.004  (-0.002, 0.011) |
| Missing ≥2 necessities | -0.036*  (-0.073, 0.002) | -0.020  (-0.054, 0.015) | 0.025  (-0.015, 0.065) | 0.023*  (-0.001, 0.048) |
| **Panel C. Individual components** |  |  |  |  |
| Missing bathroom | -0.038  (-0.083, 0.007) | 0.001  (-0.045, 0.048) | 0.008  (-0.038, 0.054) | 0.020  (-0.007, 0.047) |
| Missing heating | 0.031  (-0.021, 0.083) | -0.000  (-0.035, 0.035) | -0.022  (-0.053, 0.009) | -0.022***  (-0.039, -0.006) |
| Missing sink | -0.047**  (-0.090, -0.004) | -0.010  (-0.052, 0.031) | 0.015  (-0.030, 0.060) | 0.033**  (0.005, 0.060) |
| Missing stove/range | -0.036**  (-0.068, -0.003) | -0.004  (-0.029, 0.022) | 0.039**  (0.009, 0.069) | 0.017  (-0.006, 0.039) |
| Missing telephone | 0.014  (-0.005, 0.032) | -0.022**  (-0.040, -0.005) | 0.001  (-0.013, 0.015) | 0.009**  (0.000, 0.018) |
| Missing refrigerator | -0.042*  (-0.087, 0.003) | -0.024  (-0.071, 0.022) | 0.034  (-0.008, 0.075) | 0.022  (-0.008, 0.053) |
| **Panel D. Housing Tenure** |  |  |  |  |
| Individuals who rent | 0.33  (-1.33,1.98) | -2.12***  (-3.48, -0.75) | 1.52  (-0.23,3.27) | 0.09  (-0.89,1.07) |
| Individuals who own a  house | 4.24  (-1.72,10.20) | -2.66  (-.42,1.10) | -0.69  (-.30,0.91) | 1.34*  (-.15,2.84) |

Notes: Estimates are from survey-weighted linear probability models. Coefficients represent percentage point changes. Models include demographic controls, state fixed effects, and year fixed effects. The sample is restricted to adults aged 18–64 with income ≤138% FPL. 2014 is excluded as a transition year. *p<0.10, ** p<0.05, *** p<0.01

Table A4: Adjusted DID Estimates of 2014 Medicaid Expansion on Insurance Coverage (Ages 26–64)

|  | **Uninsured** | | **Medicaid** | | **Employer** | | **Direct** | |  |
| --- | --- | --- | --- | --- | --- | --- | --- | --- | --- |
|  | |  | |  | |  | |  | |
| **With complete basics** | | *−*7*.*02***  (-9.87,-4.17） | | 13*.*72***  (10.78,16.67) | | *−*2*.*31***  (-3.17, -1.45) | | *−*5*.*11***  (-6.83, -3.39) | |
| **Lacking ≥1 necessity** | | *−*6*.*56***  (-10.58, -2.54) | | 13*.*19***  (9.40, 16.98) | | *−*2*.*83***  (-4.58, -1.07) | | *−*5*.*90***  (-9.18, -2.62) | |

Notes: Estimates are from survey-weighted linear probability models. Coefficients represent percentage point changes. Models include demographic controls, state fixed effects, and year fixed effects. The sample is restricted to adults aged 18–64 with income ≤138% FPL. 2014 is excluded as a transition year. *∗p <* 0*.*1, *∗∗p <* 0*.*05, *∗∗∗p <* 0*.*01

1. **Event Study**

**
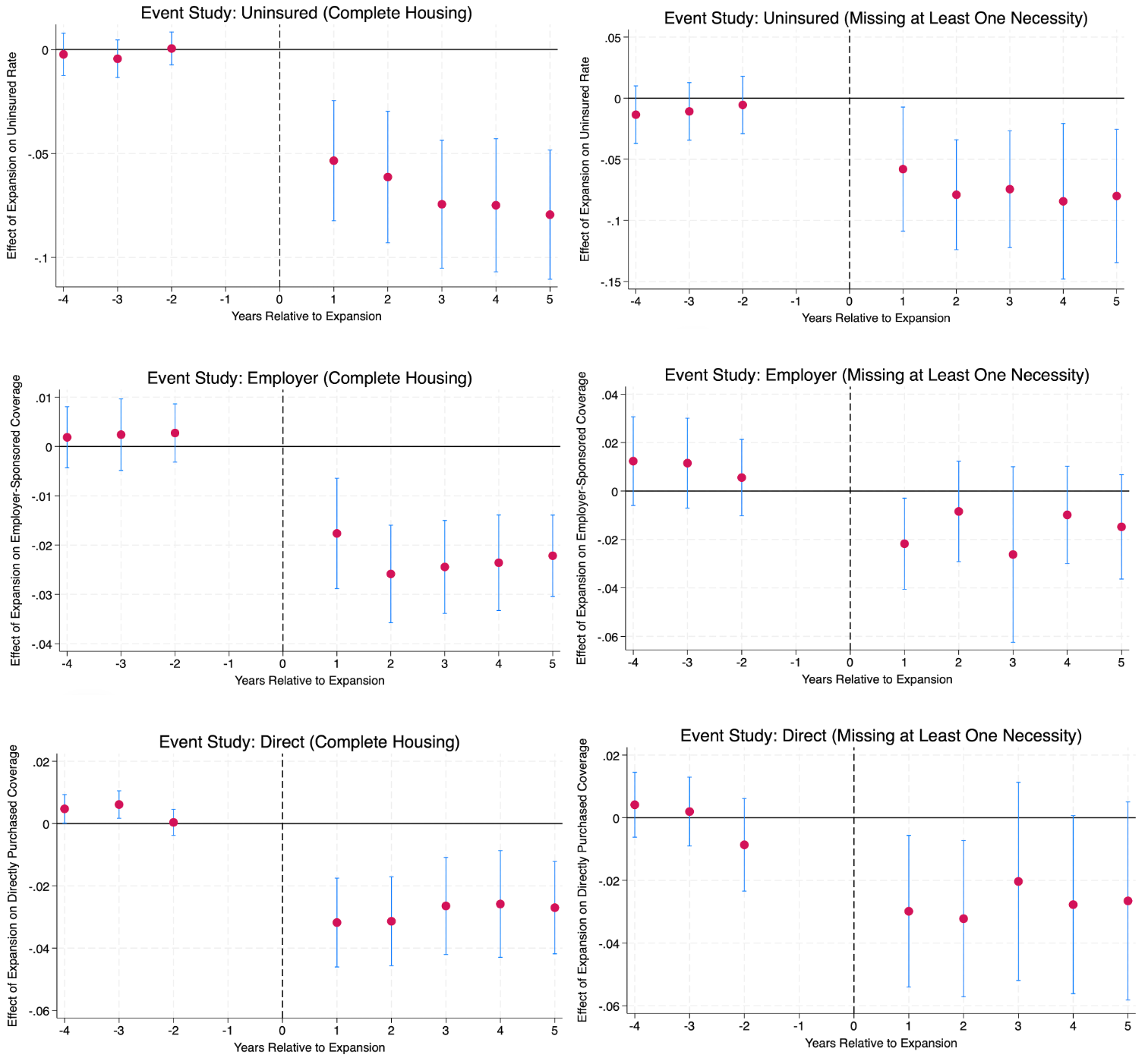
**

Figure A1| Estimates are from survey-weighted linear probability models. Coefficients represent percentage point changes relative to the year prior to expansion (t = −1). Models include demographic controls, state fixed effects, and year fixed effects. The sample is restricted to adults aged 18–64 with income ≤138% FPL. 2014 is excluded as a transition year. The vertical dashed line indicates the year of Medicaid expansion (2014). Error bars represent 95% confidence intervals.

Table A5. Event study models and linear pre-trend tests

| **Outcome** | **Housing group** | **Joint pre-trend test (p-value)** | **Linear pre-trend coefficient** | **Linear pre-trend p-value** |
| --- | --- | --- | --- | --- |
| **Uninsured** | With complete basics | 0.6124 | 0.0012 | 0.4701 |
|  | Lacking ≥1 necessity | 0.6859 | 0.0048 | 0.3008 |
| **Medicaid** | With complete basics | 0.5160 | -0.0002 | 0.9375 |
|  | Lacking ≥1 necessity | 0.9060 | 0.0017 | 0.5688 |
| **Employer-sponsored** | With complete basics | 0.8163 | -0.0005 | 0.6819 |
|  | Lacking ≥1 necessity | 0.5367 | -0.0042 | 0.2261 |
| **Directly purchased** | With complete basics | 0.0555 | -0.0020 | 0.0188 |
|  | Lacking ≥1 necessity | 0.0850 | -0.0027 | 0.0768 |

Notes: Estimates are from survey-weighted linear probability models. The table reports tests of the parallel trends assumption from event study models. The joint pre-trend test reports p-values from F-tests of the null hypothesis that all pre-expansion event-time coefficients are jointly equal to zero (years −4 to −2). The linear pre-trend test reports the coefficient and p-value from a regression of the outcome on a linear time trend interacted with expansion status using pre-expansion data (2010–2013). Event-time coefficients are normalized to the year prior to expansion (t = −1). Models include demographic controls, state fixed effects, and year fixed effects. The sample is restricted to adults aged 18–64 with income ≤138% FPL. 2014 is excluded as a transition year.
